# Cough event classification by pretrained deep neural network

**DOI:** 10.1186/1472-6947-15-S4-S2

**Published:** 2015-11-25

**Authors:** Jia-Ming Liu, Mingyu You, Zheng Wang, Guo-Zheng Li, Xianghuai Xu, Zhongmin Qiu

**Affiliations:** 1 Department of Control Science and Engineering, Tongji University, Shanghai, 201804, China; 2 Department of Respiratory Medicine, Tongji Hospital of Tongji University, Shanghai, 200065, China; 3 Data Center of Traditional Chinese Medicine, China Academy of Chinese Medical Science, Beijing, 100700, China

## Abstract

**Background:**

Cough is an essential symptom in respiratory diseases. In the measurement of cough severity, an accurate and objective cough monitor is expected by respiratory disease society. This paper aims to introduce a better performed algorithm, pretrained deep neural network (DNN), to the cough classification problem, which is a key step in the cough monitor.

**Method:**

The deep neural network models are built from two steps, pretrain and fine-tuning, followed by a Hidden Markov Model (HMM) decoder to capture tamporal information of the audio signals. By unsupervised pretraining a deep belief network, a good initialization for a deep neural network is learned. Then the fine-tuning step is a back propogation tuning the neural network so that it can predict the observation probability associated with each HMM states, where the HMM states are originally achieved by force-alignment with a Gaussian Mixture Model Hidden Markov Model (GMM-HMM) on the training samples. Three cough HMMs and one noncough HMM are employed to model coughs and noncoughs respectively. The final decision is made based on viterbi decoding algorihtm that generates the most likely HMM sequence for each sample. A sample is labeled as cough if a cough HMM is found in the sequence.

**Results:**

The experiments were conducted on a dataset that was collected from 22 patients with respiratory diseases. Patient dependent (PD) and patient independent (PI) experimental settings were used to evaluate the models. Five criteria, sensitivity, specificity, F1, macro average and micro average are shown to depict different aspects of the models. From overall evaluation criteria, the DNN based methods are superior to traditional GMM-HMM based method on F1 and micro average with maximal 14% and 11% error reduction in PD and 7% and 10% in PI, meanwhile keep similar performances on macro average. They also surpass GMM-HMM model on specificity with maximal 14% error reduction on both PD and PI.

**Conclusions:**

In this paper, we tried pretrained deep neural network in cough classification problem. Our results showed that comparing with the conventional GMM-HMM framework, the HMM-DNN could get better overall performance on cough classification task.

## Background

Cough is a common symptom in respiratory diseases. It's a protective reflex that helps the human body to exhale secretion from the airways, protecting the lower airways. In the treatment of cough related diseases, cough severity is an essential factor in monitoring the progression of diseases. In modern clinical practice, the measurement of cough severity is mainly based on self report scales such as cough scores, visual analogue scales (VAS) and quality-of-life questionnaires [1]. These scales gather information from patients' subjective feeling of cough severity. Though being wildly used, these scales are still rough, because the subjective scales are easily influenced by patients' own tolerance and placebo effect. The solution for objectively measuring cough severity can be an accurate cough monitor device, which can record the information of cough objectively. Beside evaluating cough severity and therapeutic efficacy, such devices could also guide clinical practice and research in chronic cough [2,3].

Cough detection system is an essential component of such cough monitor devices. The early attempt of cough detection system is to hire a human expert to manually record patients' cough from their audio and video monitor [4]. The cost is so high as to make it barely useful. In recent years, automatic cough detection methods has been developed to cut the cost for detecting cough.

Researchers have tested different pattern recognition techniques for the cough detection problem. S. Barry et. al. [5] proposed a framework for cough counter with probability neural network as classifier. In their work, mel frequency cepstral coefficients and linear predictive cepstral coefficients were extracted on frame level. Before feeding the features into probability neural network, principle component analysis was employed to reduce the feature dimension. They also used an energy threshold based audio segment algorithm to segment continuous audios into samples. After getting the predicted label for each frame, a final decision for each sample would be made by calculate the domestic label in that segmentation. They tested this farmework in a 1 hour audio dataset, and achieved 80% sensitivity and 96% specificity. S. Matos et. al. [6] presented an HMM based cough event detection algorithm. 39D MFCC (MFCC+Δ+Δ) was used as feature representation for each frame. This work uses three HMMs with different state number to fit cough samples and 128 HMMs with 3 states each to fit non cough parts, where the 128 HMMs was called "filler models". After all the HMMs being trained, a decoding process was followed to find the most likely path, from which the cough parts could be located. They also employed an energy threshold based filtering strategy to discard low energy samples. In the high energy audio parts, the whole algorithm achieved an average 85% sensitivity and almost 100% specificity. They further extended the previous work by adding a classification step after the event detection step, build a 24-h cough monitor, LCM [7]. E. Larson et. al. [8] used random forest classifier to recognise the explosive sounds in cough signals. Their features come from PCA decomposed raw spectrogram. When getting the PCA projection, only the spectrograms from explosive sounds were used. With this configuration, the overall system achieved 92% precision and false positive rate of 0.5% in a 72 h dataset. S. Larson et. al. [9] employed support vector machine to detect cough. Continuous audio streams were first segmented by an energy threshold based event detection algorithm. In each segmented event, each frame was then classified by SVM, and the output of the frames were counted. The label of the event was labeled as cough if more than 1/3 of the frames were classified as cough. They collected 25.5 hours of audio data and 75.5% of the cough episodes were detected with 99.6% specificity.

Though there are many systems have been developed, there are still no golden standard in cough detection task. The main problem is the unsatisfactory detection precision. Meanwhile, most of the works have not been validated in large scale, leading to unconvincing results. The most prevalent system, LCM [7], finds a tradeoff between performance and human labor. In their classification step, they employ human experts to label all the detected cough events from a detection step, the labeled coughs and noncoughs are fed into an HMM-GMM model for further classification. The performance of this work reached basic requirement for real world application. However, the performance of HMM-GMM models could be further improved from the recent years breakthrough in the field of automatic speech recognition [10,11] by replacing GMM with a newly developed model, deep neural network (DNN), to model observational probability of HMMs. By training the discriminative neural network models for the posterior probability, information from each frame could be better learned. The benefit of using a neural network has been known for a long time, but it's not feasible to train a deep neural network until the recent introduction of pretrain strategy. As for cough classification problem, it is natural to transfer the classification models into deep neural networks. DNN is a model with more powerful learning ability that could replace GMM when modeling cough classifiers. Raw signals for cough classification are audio recordings, which is naturally modelled by deep neural networks. Moreover, the cough patterns and non-cough patterns are also varied in a large range, which requires a more capable model to learn the variances. In this paper, we proposed a pertrained deep neural network based framework for cough classification.

## Methods

### Pretrained deep neural networks

Previously, the obstacles from training a deep neural network mainly consisted in two aspects. One is the low computational resource at that time. The other is the vanishing gradient problem that being prevent the lower level weights from being properly trained. The first obstacle is solved by the development of computational equipment. The second is partly solved by the introduction of pretrain strategy with Deep Belief Network (DBN), which is composed with multilayer stacked Restricted Boltzmann Machines.

### Restricted Boltzmann Machine

**Figure 1:**
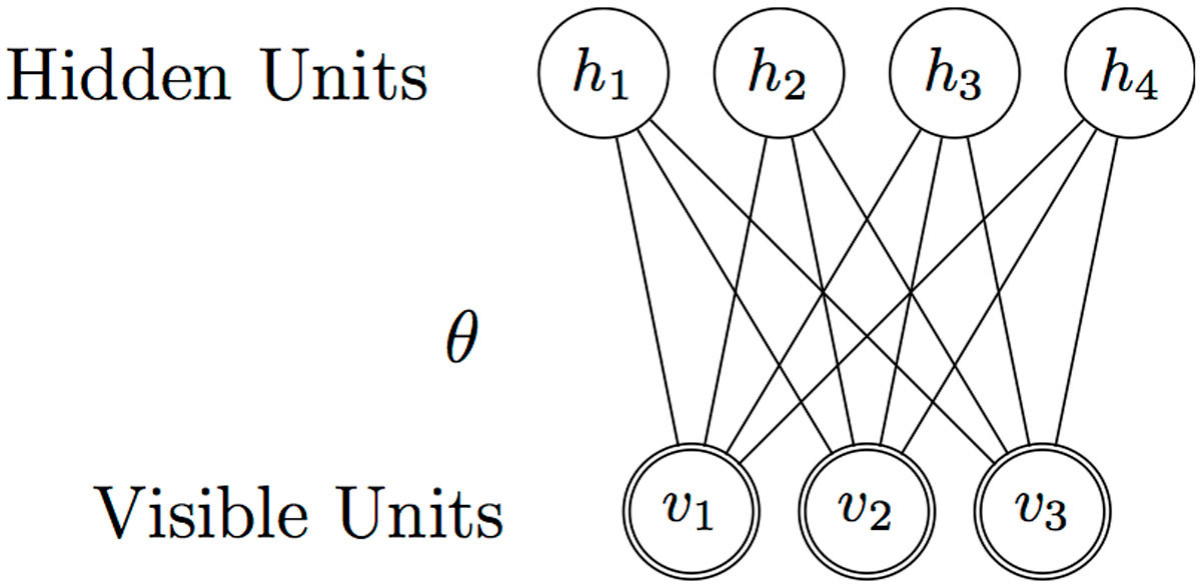
**A simple example of RBM with 3 visible units and 4 hidden units**.

Restricted Boltzmann Machine (RBM) is a stochastic neural network. The basic presumption of RBM is that the features we observed are controlled by many high level factors, so that the high level factors could be used as features with higher level abstraction. It's also a kind of log-linear Markov Random Field. The visible units **v **represent the raw feature vector, where *v_j _*is the value of *i*th feature. The higher level factors are encoded in vector **h**, where *h_i _*is the *i*th factor. Entries of RBMs are connected via weights. Intuitively, weights identify the correlation of the nodes. Larger weight means larger possibility that the connected nodes would cooccur. In RBM, connections are only allowed between visible unites and hidden units, which is the reason why it's called "restricted". A simple example of RBM is displayed in Figure 1.

(1) $$p\left(v,h;\theta \right)=\frac{1}{Z\left(\theta \right)}e^{-E\left(v,h;\theta \right)}$$

(2) $$Z\left(\theta \right)=\underset{u}{\sum }\underset{h}{\sum }e^{-E\left(u,h\right)}$$

The nodes of RBMs are associated with different assumptions to fit different problems. As Markov Random Fields, there are different potential energy functions that based on different assumptions. For each configuration of all the nodes, the possibility of that configuration is further determined by the potential energy function by dividing the partition function as Equ. 1 and Equ. 2, where **u **and **h **represents all the possible configurations of both viable and hidden units. In binary RBMs, both the visual nodes and the hidden nodes are with binary values. The potential energy function is defined as Equ. 3. The joint probability could be easily derived from Equ. 1 and Equ. 2.

(3) $$E\left(v,h;\theta \right)=-\overset{V}{\underset{i=1}{\sum }}\overset{V}{\underset{j=1}{\sum }}W_{ij}v_{i}h_{j}-\overset{V}{\underset{i=1}{\sum }}b_{i}v_{i}-\overset{H}{\underset{j=1}{\sum }}a_{j}h_{j}$$

With the joint probability, we could further derive marginal probability distribution assigned to a visible vector **v **by marginalise **h**. The marginal distribution is shown in Equ. 4. The parameters of the RBM could then be trained by mini-batch gradient descent. The derivatives of the log likelihood function with respect to the parameters **W**, **v **and **h **is shown in Equ.5, 6 and 7.

(4) $$p\left(v|\theta \right)=\frac{1}{Z\left(\theta \right)}\underset{h}{\sum }e^{-E\left(v,h\right)}$$

(5) $$\frac{\partial logP\left(M;\theta \right)}{\partial W}=\underset{v\in M}{\sum }\left(E_{P\left(h|v\right)}\left[vh^{T}\right]-E_{P\left(h,v\right)}\left[vh^{T}\right]\right)$$

(6) $$\frac{\partial logP\left(M;\theta \right)}{\partial a}=\sum_{v\in M}\left(E_{P\left(h|v\right)}\left[h\right]-E_{P\left(h,v\right)}\left[h\right]\right)$$

(7) $$\frac{\partial logP\left(M;\theta \right)}{\partial b}=\underset{v\in M}{\sum }\left(E_{P\left(h|v\right)}\left[v\right]-E_{P\left(h,v\right)}\left[v\right]\right)$$

where *M *is the mini-batch data set. *E_P _*means the expectation w.r.t. the distribution *P*. *P*(**h**|**v**) is the distribution of the hidden units given the observed visible vector, and *P*(**h**, **v**) is the joint distribution of the hidden and visible units. The conditional probability *P*(**h**|**v**), and *P*(**h**|**v**), are depicted as Equ. 8 and Equ. 9, where *Ber*(*x*). From the distributions we could find the expectations of conditional probabilities are easy to compute. However, in order to calculate the expectation of the joint probability *P*(**h**, **v**), we need to use Gibbs sampling starting from a random vector and sample hidden units and visible units repeatedly for a long time. To boost the calculation of the gradients, an process named "contrastive divergence (CD)"[12] could be used to estimate the gradients by replace joint expectation by the "reconstruction" of the hidden and visible units. The general process of CD is to set a data vector **v **as the initial point of a sampler before recursively sampling hidden units and visible units based on the conditional distribution. Usually, the **v **and **h **after sampling once are good enough to update parameters, and this setting is named as CD_1_. This method is much more efficient than a naive Gibbs sampler.

(8) $$p\left(h|v;\theta \right)=\underset{j}{\prod }P\left(h_{j}|v\right)=\underset{j}{\prod }Ber\left(h_{j}|\delta \left(\underset{i}{\sum }W_{ij}v_{i}+a_{i}\right)\right)$$

(9) $$p\left(v|h;\theta \right)=\underset{j}{\prod }P\left(v_{j}|h\right)=\underset{j}{\prod }Ber\left(v_{j}|\delta \left(\underset{i}{\sum }W_{ij}h_{i}+b_{i}\right)\right)$$

The units of the RBMs could associate with different type of values. For real value data, Gaussian-Bernoulli RBM is the model that always employed.

The potential energy function of a Gaussian-Bernoulli RBM is shown in Equ.10. We could easily get the probability distributions for a Gaussian-Bernoulli RBM with the energy function follow the same steps as binary RBM. *P*(**h**|**v**) is in a different form from binary RBM, which is shown in Equ.10.

(10) $$E\left(v,h;\theta \right)=-\overset{D}{\underset{i=1}{\sum }}\overset{F}{\underset{j=1}{\sum }}W_{ij}h_{j}\frac{v_{i}}{\sigma_{i}}-\overset{D}{\underset{i=1}{\sum }}\frac{{\left(v_{i}-b_{i}\right)}^{2}}{2\sigma_{i}^{2}}-\overset{F}{\underset{j=1}{\sum }}a_{j}h_{j}$$

(11) $$p\left(v|h;\theta \right)=\underset{i}{\prod }N\left(v_{i}|\sigma \underset{j}{\sum }W_{ij}h_{j}+b_{j},\sigma \right)$$

The update rule can be easily derived follow the exact same steps for binary RBMs. CD_1 _is also an efficient method for training a Gaussian-Bernoulli RBM.

### Deep Neural Network

In the Deep Neural Network (DNN) prototype, RBMs are used to learn the initialisation of the DNN. A multilayer RBM, which is also named Deep Belief Network (DBN), could be trained by a greedy approach. In the first layer, the data vectors are used to train the first layer RBM. After well trained, the expectations of the hidden units are used as the data vectors for the second layer RBM. The upper layers of the RBMs are all trained with this process. After all, the stacked RBMs form a DBN. The training steps for DBN are also shown in Table 1 where N refers to the depth of the DBN.

**Table 1:** Algorithm description of train deep belief network.

Input: Data D = {*x*}, desired layers *K *and nodes number for each layer *N*_*i*_
Output: The structure and learned initialization parameters of the DNN.
1. Learn parameters *θ*_1 _for the 1*^st ^*layer RBM from data.
For k = 2:*K*
2. Initialize the k-th layer RBM by unroll the k-1th layer RBM to the kth layer, of which parameters $W_{k}=W_{k-1}^{T}$
3. Refine the parameters of kth layer RBM from data vectors generated from k-1th layer.
Return: Structure and parameters of the stacked RBMs.

Greedy training process for deep belief network

After training the DBN, the parameters of it are copied to a neural network the same structure. The output layer of the DNN is a softmax layer which stacked on the top of the network. The process that trains the DBN and copies its parameter is called "pretrain". The initialised neural network is further trained by conventional Back Propagation algorithm. This further training step is called "fine-tuning". The pretrain step helps DNN in several ways. It's force the DNN to map the raw feature space to higher abstraction level, not just to learn a scalar response to the output space, which actually acts like a data-induced regularizer. It also helps BP to learn a better local minima which has better generalisation properties [13,14].

Though the DNN could be well trained with previous process, DNN could not deal with temporal data well by itself. When dealing with audio data, we combine DNN and HMM to generate a better decoder. Raw features from a frame and its consecutive frames are sent to DNN together, in which way the dependence of consecutive frames can be learnt by the DNN. Each output unit represents a HMM state, which is to say the DNN predicts the observation probabilities that current frame belongs to each state. Three cough HMMs and one noncough HMM are trained from the training set. After the observation probabilities are calculated, a viterbi decoding algorithm would be applied with all of the HMMs. For each sample, a transcription, which contains the most likely HMM decoding sequence, could be generated from the decoding process. A sample is labeled as cough if a cough HMM is found in the transciption sequence.

**Figure 2:**
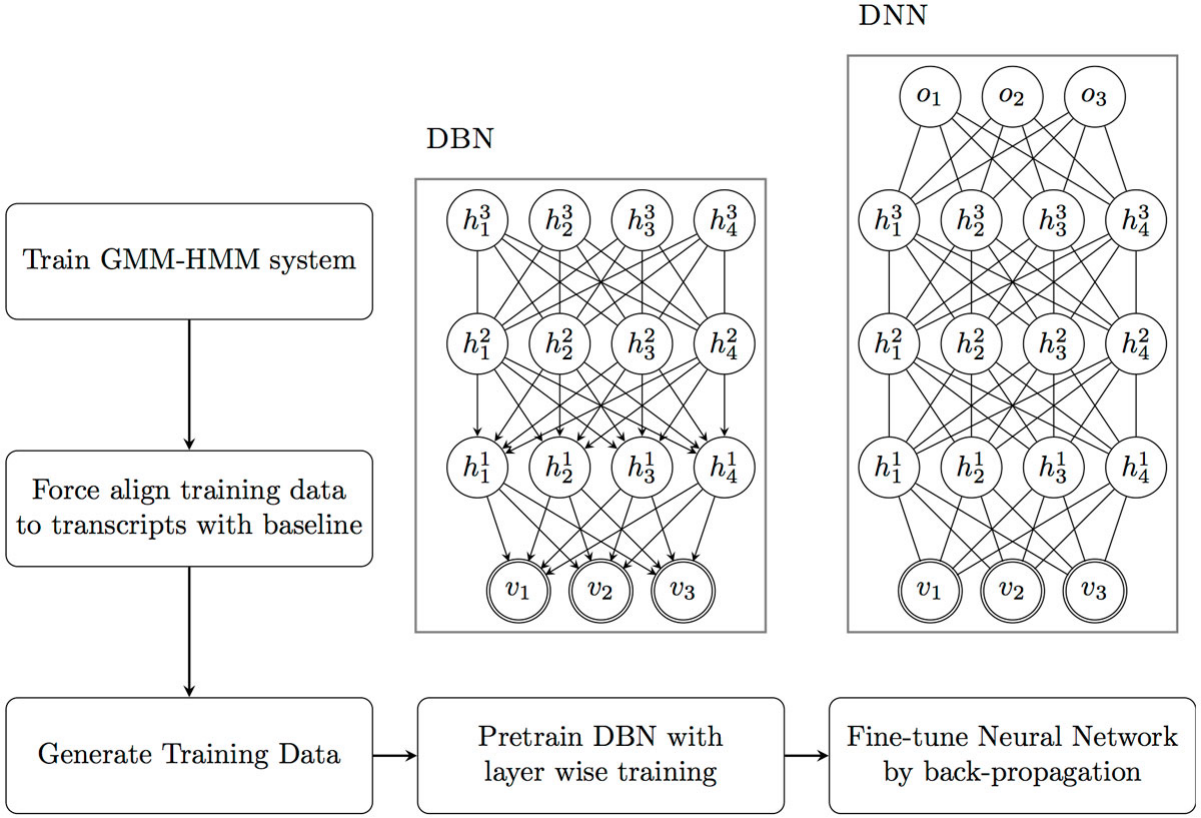
**The training process of combination of DNN and HMM**.

The "ground truth" for states is generated by employing a GMM-HMM baseline system to transcript the training data beforehand. The raw features are conventional 39-D MFCC. In the prototype of combining DNN and HMM, DNN is used to calculate observation probebility, and HMM is used to decode the temporal structure. The training process for a DNN-HMM system is shown in Figure 2.

### Feature representation

The raw feature extracted in this work was 39D Mel Frequency Cpestral Coefficients (MFCC). MFCC is a wildly used feature set in audio analysis works. Firstly, after pre-emphasis, Fourier Transform was applied on windowed signal to compute the power spectrum. Then, the power spectrum was mapped on mel scale with Mel filter banks. We got the logarithm of the power output of each Mel filter. Discrete cosine transform (DCT) was then applied on the log powers. A further normalization process named lifting was usually employed. The lifting process is given as Eq. 12. We set *L *= 22 and *N *= 40. In this paper, first 13 MFCC and their first and second-order derivatives were included in experiments. The parameters for window was as the conventional configuration for audio signal analysis. Here 25 ms Hamming window was employed with a 15 ms frame overlap.

(12) $$c^{\prime }\left(n\right)=\left(1+\frac{L}{2}\text{sin}\left(\frac{n\pi }{L}\right)\right)c\left(n\right)$$

### Data collecion

The raw audio recordings are collected from 22 patients in China who were suffering various respiratory diseases, 12 community-acquired pneumonia(CAP) patients, 2 bronchial asthma(BA) patients and 8 chronic obstructive pulmonary disease(COPD) patients. This study was approved by the Ethics Committee of Tongji Hospital and registered with Chinese Clinical Trials Register (http://www.chictr.org.cn/index.aspx) number ChiCTR-ONC-08000152. and all patients gave informed consent. The recording system was constituted by a portable digital audio recorder (SONY ICD-LX30) and a microphone (ECM-CS10), and attached on patients' collar. Recording lasted for about 24 hour on each patient. Recording quality was configured at 44.1 kHz sampling frequency and 192 kbps bit rate. All patients were encouraged to ignore the recording system and perform their daily activities, so that we could record sounds in natural hospital environment which is the environment this system will be deployed.

The raw data were segmented into 10-min clips, and labeled by two annotators. The annotation process was based on Pratt platform[15]. The starting and ending of each individual cough was marked. The definition of "cough" here is "cough sounds" by[16]. After labelling, each 10-min clips were further segmented into 10-s clips.

### Data preparation with a event detection system

In this step, we employed a conventional keyword spotting method for cough detection[6]. This algorithm employs few HMMs to model cough signal, and lot of HMMs to model the other audio signals. In this paper, we used 3 cough models and 128 filler models as the authors recommanded.

Our main concern in this step is to find most of the coughs. Thus we randomly reduce the non-cough data to underfit the filler models. The number of non-cough segments was set as twice as the segments that contain cough. With this setting, the confidence of non-cough signals are manually suppressed, making many low confidential cough signals detected. Meanwhile, reducing the amount of data significantly reduced the training time.

After well trained, the detection model was applied to decode on training set. All the segments that label as cough by the model were further cut to isolated sounds for classification step. All the detected cough intervals was segmented into individual samples. After that, each sample was examined by an annotator, getting its label as cough or noncough. All the isolated samples were fed into the DNN-HMM classification model. The samples are considered positive if there are cough labels in the transcription after classification, and negative is the samples that there are no cough labels or no valid labels in the transcription.

## Results

### Evaluation setup

For training the DBN model, the mini-batch size of mini-batch stochastic gradient decent was set to 256. The Gaussian-Bernoulli RBMs was trained with maximum of 200 training epochs. And the maximum number of epochs for other binary RBMs was set to 100. We used fixed learning rate which was 0.01. In fine-tuning step, the mini-batch size was also 256, and the learning rate was 0.008.

The detection system was developed on the basis of HTK toolkit [17]. The classification step was implemented on Kaldi toolkit [18]. We run the experiments on a computational server which contains 33 GB Memory, Intel(R) Xeon(R) CPU E5-2603 (1.8 GHz/8-core) and a NVIDIA Tesla K20 GPU with 5 GB of GDDR5 RAM and 2496 processing cores.

For results evaluation, we used five criteria, sensitivity, specificity, F1 measure, macro average and micro average. When evaluating classification performance, the core test sets came from two sources, a patient dependent test set and a patient independent test set. In the patient dependent test set, 16 patients out of the whole patient set were selected. From which 2/3 of the 10-s audio segmentations were selected as training set, the rest were test set. In the patient independent setting, all the 10-s clips in the other 6 patients would be used as test set. The number of the samples in each source is listed in Table 2.

**Table 2:** Number of data in each set.

	Training set	Patient dependent test set	Patient independent test set
Cough Samples	3873	3119	1742

Noncough Samples	2347	18862	8161

### Experiment results

**Figure 3:**
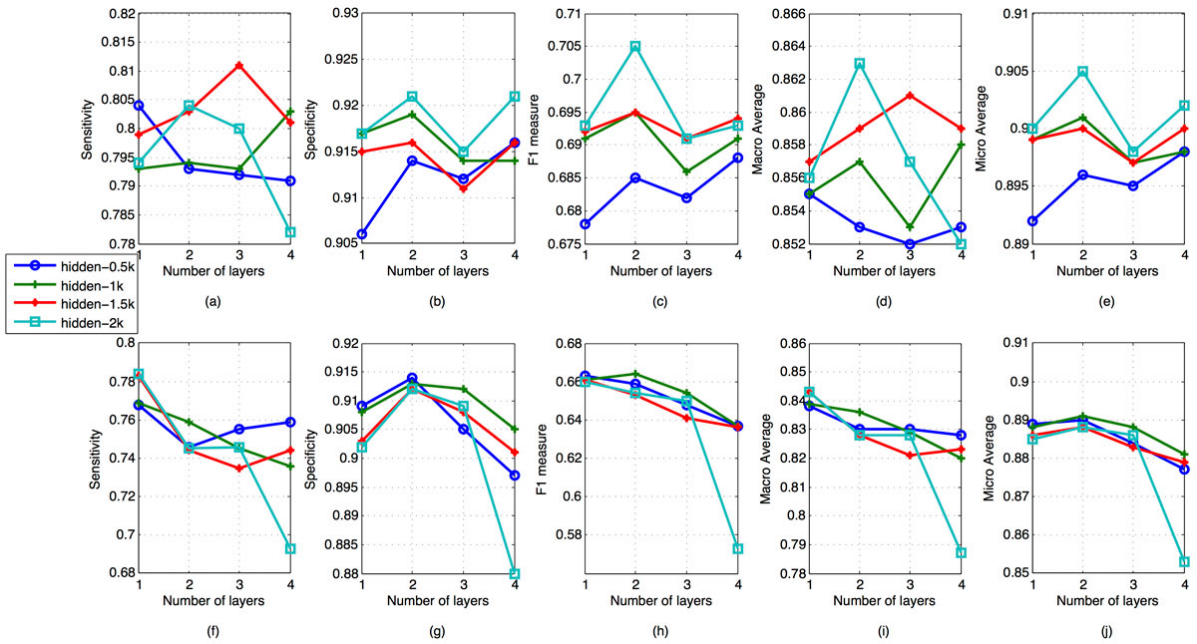
**Performances on patient dependent test set**. The results are shown as a function of the number of layers and number of hidden units in each layer. The performance of baseline was generated from a conventional GMM-HMM model.

The results that earned from patient dependent test set is shwon in Figure 3, Table 3 and Table 4. In Figure 3, the above pictures showed the results from pretrained deep neural networks and the below pictures from neural networks that were randomly initialized. The bold numbers in the tables are the best results in all of the DNN models. Overall, without pretrain step, the neural network can only surpass the baseline on F1 and micro average. After the pretrain step added, the DNN based models reached the same level of macro average as baseline, and they are also superior than baseline on F1 and micro average. The best results for F1, macro average and micro average achieved by DNNs are 0.705, 0,863, and 0.905 respectively, the baseline counterparts are 0.563, 0.856, 0.792 respectively. In sensitivity, the baseline outperforms all of the DNN models. At the same time, all of the DNN models outperform the baseline by a large extend. We get 14% error reduction on F1 and 11% error reduction on micro average on this test set.

**Table 3:** Result table with pretrained neural network on PD.

# layers	# hidden units	**Sens**.	**Spec**.	F1	**Mac. Ave**.	**Mic. Ave**.
1	512	0.804	0.906	0.678	0.855	0.892

1	1024	0.793	0.917	0.691	0.855	0.899

1	1536	0.799	0.915	0.692	0.857	0.899

1	2048	0.794	0.917	0.693	0.856	0.9

2	512	0.793	0.914	0.685	0.853	0.896

2	1024	0.794	0.919	0.695	0.857	0.901

2	1536	0.803	0.916	0.695	0.859	0.9

2	2048	0.804	**0.921**	**0.705**	**0.863**	**0.905**

3	512	0.792	0.912	0.682	0.852	0.895

3	1024	0.793	0.914	0.686	0.853	0.897

3	1536	**0.811**	0.911	0.691	0.861	0.897

3	2048	0.8	0.915	0.691	0.857	0.898

4	512	0.791	0.916	0.688	0.853	0.898

4	1024	0.803	0.914	0.691	0.858	0.898

4	1536	0.801	0.916	0.694	0.859	0.9

4	2048	0.782	0.921	0.693	0.852	0.902

Baseline Method		0.945	0.767	0.563	0.856	0.792

**Table 4:** Result table with randomly initialized neural network on PD.

# layers	# hidden units	**Sens**.	**Spec**.	F1	**Macro Ave**.	**Mic. Ave**.
1	512	0.768	0.909	**0.663**	0.838	0.889

1	1024	0.769	0.908	0.661	0.839	0.888

1	1536	0.783	0.903	0.661	0.843	0.886

1	2048	**0.784**	0.902	0.66	**0.843**	0.885

2	512	0.746	**0.914**	0.659	0.83	0.89

2	1024	0.759	0.913	0.664	0.836	**0.891**

2	1536	0.744	0.912	0.653	0.828	0.888

2	2048	0.745	0.912	0.654	0.828	0.888

3	512	0.755	0.905	0.648	0.83	0.884

3	1024	0.745	0.912	0.654	0.829	0.888

3	1536	0.735	0.908	0.641	0.821	0.883

3	2048	0.746	0.909	0.65	0.828	0.886

4	512	0.759	0.897	0.637	0.828	0.877

4	1024	0.736	0.905	0.637	0.82	0.881

4	1536	0.744	0.901	0.636	0.823	0.879

4	2048	0.693	0.88	0.573	0.787	0.853

**Figure 4:**
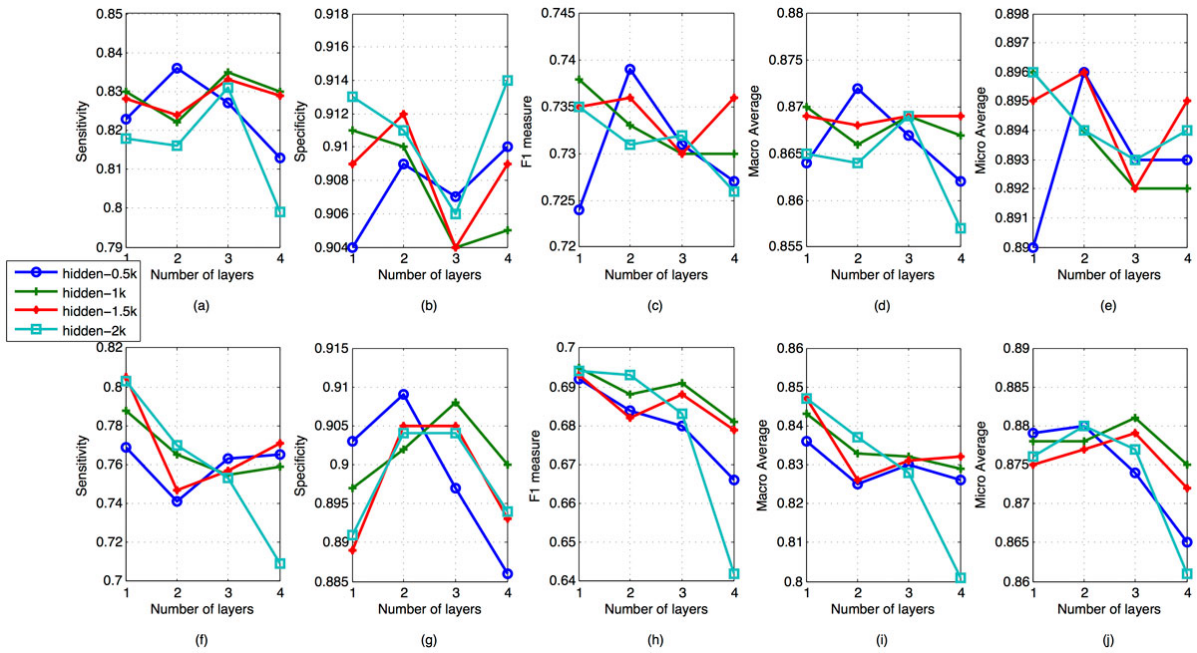
**Performances on patient independent test set**. The setting here is as same as Figure 3, except that these results are generated from a patient independent test set.

Similar results could be found in patient independent experiments in Figure 4, Table 5 and Table 6. The performance of the baseline method is also given in Table 3 and 5. The randomly initialized neural networks still could outperform baseline on macro average. Pretrained deep neural network models outperform baseline on specificity, F1 and micro average, meanwhile achieve the similar level on macro average. The value of these metrics form baseline are 0.632, 0.868, 0.801 respectively on this test set, and that from best DNNs are 0.705, 0.863 and 0.905. The error reduction is 7% and 10% on F1 and micro average respectively.

**Table 5:** Result table with pretrained neural network on PI.

# layers	# hidden units	**Sens**.	**Spec**.	F1	**Macro Ave**.	**Mic. Ave**.
1	512	0.823	0.904	0.724	0.864	0.89

1	1024	0.83	0.911	0.738	0.87	**0.896**

1	1536	0.828	0.909	0.735	0.869	0.895

1	2048	0.818	0.913	0.735	0.865	**0.896**

2	512	**0.836**	0.909	**0.739**	**0.872**	**0.896**

2	1024	0.822	0.91	0.733	0.866	0.894

2	1536	0.824	0.912	0.736	0.868	0.896

2	2048	0.816	0.911	0.731	0.864	0.894

3	512	0.827	0.907	0.731	0.867	0.893

3	1024	0.835	0.904	0.73	0.869	0.892

3	1536	0.833	0.904	0.73	0.869	0.892

3	2048	0.831	0.906	0.732	0.869	0.893

4	512	0.813	0.91	0.727	0.862	0.893

4	1024	0.83	0.905	0.73	0.867	0.892

4	1536	0.829	0.909	0.736	0.869	0.895

4	2048	0.799	**0.914**	0.726	0.857	0.894

Baseline method		0.971	0.765	0.632	0.868	0.801

**Table 6:** Result table with randomly initialized neural network on PI.

# layers	# hidden units	**Sens**.	**Spec**.	F1	**Mac. Ave**.	Mic. Ave.
1	512	0.769	0.903	0.692	0.836	0.879

1	1024	0.788	0.897	**0.695**	0.843	0.878

1	1536	**0.805**	0.889	0.693	**0.847**	0.875

1	2048	0.803	0.891	0.694	**0.847**	0.876

2	512	0.741	**0.909**	0.684	0.825	0.88

2	1024	0.765	0.902	0.688	0.833	0.878

2	1536	0.747	0.905	0.682	0.826	0.877

2	2048	0.77	0.904	0.693	0.837	0.88

3	512	0.763	0.897	0.68	0.83	0.874

3	1024	0.755	0.908	0.691	0.832	**0.881**

3	1536	0.757	0.905	0.688	0.831	0.879

3	2048	0.753	0.904	0.683	0.828	0.877

4	512	0.765	0.886	0.666	0.826	0.865

4	1024	0.759	0.9	0.681	0.829	0.875

4	1536	0.771	0.893	0.679	0.832	0.872

4	2048	0.709	0.894	0.642	0.801	0.861

## Discussion

In independent test set, the test patients don't appear in the training set, and the models achieved similar results as in patient dependent test set. That shows the models have certain generalization ability in cough classification task. The models that trained on some patients could also capture the general characteristics of cough and noncough, not just overlearn certain parterns from the training patients's data.

In order to examine the effieciency of the pretrain step, the performances of pretrained and randomly initialized conventional neural networks are compared in the same setting. In all of the structures, pretrained Deep Neural Networks always perform better than the counterpart. In macro average, the worst cases from pretrained Deep Neural Network are 0.852 and 0.857 for patient dependent and independent test set, while the best cases for conventional neural network are 0.843 and 0.847. Even the worst cases from pretrained Deep Neural Network are better than the best cases of a conventional neural network. In other metrics, though it doesn't show such gap, it could also show the advantage of the pretrain step.

In Figure 3 and 4, the specificity of most of pretrained DNNs increase when the second hidden layer is added, and decrease after the third layer is added. When training the forth layer however, the specificity grow again. The trend of sensitivity is almost the reverse of specificity. In PD set, the sensitivity and specificity of a two layer pretrained DNN all outperform that of one layer with 2 k nodes for each layer. This also lead to the peak value of all of F1, macro average and micro average. In PD, all of the overall evaluation criteria tend to increase with the number of nodes increasing. But the overall criteria in PI set don't share the same trend. In PI set, it's the two layer network with 0.5 k nodes that gives peaks in four criteria. These findings may reflect that a two layer neural network is enough to separate cough from noncough given the amount of training data. The reason why the best performed model came from the most complex model maybe becaue of our insufficient training data. Considering the number of free parameters in DNN models, it needs more data to train a deeper model with good generalization ability. Without pretraining step, the performance of neural networks even became worse when more layers were added. This could be clearly found with 2 k nodes neural networks in the below charts in Figure 3 and 4. Therefore, in case of the data size is not so big, pretrain is a useful technique that helps neural networks reaching better classification performance.

When compared with GMM-HMM model, the pretrained Deep Neural Networks could always get better or similar overall performances, e.g. F1, macro average and micro average. We could saftly say that this pretrained Deep Neural Network method is appealing to those care about overall performance. We also find that the pretrained DNN based model could achieve more than 90% on specificity, meanwhile the GMM-HMM model could only get about 77%. However, on sensitivity, none of the pretrained DNN based model reached 85%, meanwhile GMM-HMM model achieved 94% and 97% on PD and PI respectively. This phenomenon may probabily caused by the variance of samples in the training set. In general, coughs are more consistent than the non coughs. Therefore, GMM could learn the cough models better than the noncough models, making the likelihood of noncoughs smaller than that of coughs. So even the cough samples with low likelihood could be recognized. As for neural networks, they learned more of non cough samples and raise the likelihood of non cough samples. So they made more balanced decisiones in the final.

From this aspect, we recommand the pretrain DNN based model if there is not enough human resources to correct prediciton label. Otherwise, a GMM-HMM model could be used to classify coughs from noncoughs, and the samples that are classified as cough should be filtered by human annotator to eliminate the false positives. Considering the advantages of the two models are different, the ensemble of them may gives us more appealing classification accuracy.

## Conclusions

In this paper, a novel machine learning model, deep neural network and the pretrain step, is tested on cough classification task. The experiments were conducted on both patient dependent and patient independent test sets. On both sets, sensitivity, specificity, F1 measure, macro average and micro average were used as evaluation criteria. According to these criteria, HMM-DNN framework performs better than the conventional GMM-HMM model.

Our work here mainly forcuses on cough classification task. The cough detection task is also very essential in real world cough detection problem. In the furture, we will focus on build more powerful tools for cough detection. Meanwhile, a more stable audio recording system will be developed in order to gether clearner data. More cough data need to be continuously collected, but the considerable workload of labeling cough keeps too high to keep the pace with data collection. Thus, an efficient labeling workflow should be developed, or the lack of labeled data impedes not only the detection model, but also clinical validation.

## Competing interests

The authors declare that they have no competing interests.

## Authors' contributions

Dr. Mingyu You and Dr. Guo-Zheng Li were in charge of design and coordination of the program, review, and correction of the manuscript. Jia-Ming Liu and Zheng Wang took part in the experiment implementation and writing of the manuscript. Dr. Xianghuai Xu and Dr. Zhongmin Qiu were in charge of the collection of patients and review of the manuscript.

## Declarations

Publication costs for this article were funded by Natural Science Foundation of China under grant nos. 61273305 and 81170079 as well as the Fundamental Research Funds for the Central Universities.

This article has been published as part of *BMC Medical Informatics and Decision Making *Volume 15 Supplement 4, 2015: Selected articles from the IEEE International Conference on Bioinformatics and Biomedicine (BIBM 2014): Medical Informatics and Decision Making. The full contents of the supplement are available online at http://www.biomedcentral.com/bmcmedinformdecismak/supplements/8/S4.
